# AA-NAT, MT1 and MT2 Correlates with Cancer Stem-Like Cell Markers in Colorectal Cancer: Study of the Influence of Stage and p53 Status of Tumors

**DOI:** 10.3390/ijms18061251

**Published:** 2017-06-11

**Authors:** Jorge Casado, Almudena Iñigo-Chaves, Sergio M. Jiménez-Ruiz, Sandra Ríos-Arrabal, Ángel Carazo-Gallego, Cristina González-Puga, María Isabel Núñez, Ángeles Ruíz-Extremera, Javier Salmerón, Josefa León

**Affiliations:** 1 Biosanitary Research Institute, ibs.Granada, 18012 Granada, Spain; jorgecasado@ugr.es (J.C.); sergiojr_84@hotmail.com (S.M.J.-R.); sandrariosarrabal@hotmail.com (S.R.-A.); angel_carazo@yahoo.es (Á.C.-G.); isabeln@ugr.es (M.I.N.); arextrem@ugr.es (Á.R.-E.); fsalmero@ugr.es (J.S.); 2 Clinical Management Unit of Digestive Disease, San Cecilio University Hospital, 18012 Granada, Spain; alinch.2005@gmail.com; 3 Department of Radiology and Physical Medicine, University of Granada, 18012 Granada, Spain; 4 General Surgery Unit, San Cecilio University Hospital, 18012 Granada, Spain; crisgona2@hotmail.com; 5 Ciber of Hepatic and Digestive Diseases (CIBERehd), Biosanitary Research Institute, ibs.Granada, 18012 Granada, Spain; 6 Pediatric Unit, Granada University and San Cecilio University Hospital, 18012 Granada, Spain

**Keywords:** colorectal cancer, melatonin, cancer stem cells, p53, metastasis

## Abstract

The characterization of colon cancer stem cells (CSCs) may help to develop novel diagnostic and therapeutic procedures. p53 loss increases the pool of CSCs in colorectal cancer (CRC). Recent reports suggest that the oncostatic effects of melatonin could be related to its ability to kill CSCs. Although there are no data linking the loss of p53 function and melatonin synthesis or signaling in cancer, melatonin does activate the p53 tumor-suppressor pathway in this disease. In this work, we analyze whether the expression of melatonin synthesis and signaling genes are related to the expression of CSC markers and the implication of p53 status in samples from patients with CRC. Arylalkylamine N-acetyltransferase (AA-NAT), MT1, and MT2 expression decreased in tumor samples versus normal mucosa samples in mutated p53 (mtp53) tumors versus those with wild-type p53 (wtp53). Further, AA-NAT and MT2 expression were lower in advanced stages of the disease in wtp53 tumors. On the contrary, CD44 and CD66c expression was higher in tumor versus normal mucosa in wtp53 tumors. Additionally, CD44 expression was higher in advanced stages of the disease regardless of the p53 status. Patients with CD44_high_CD66c_high_ and wtp53 tumors in advanced stages showed low expression of AA-NAT and MT2 in wtp53 tumors. These results could indicate a possible interaction of these pathways in CRC.

## 1. Introduction

Colorectal cancer is the third most common cancer in men and the second most common in women, and this disease is a major cause of death worldwide [1]. In the last decade, there have been important advances in the knowledge of molecular and genetic alterations responsible for the development of colorectal cancer; however, at present there are only a few predictive biomarkers available to select patients who would respond better to the currently available high-cost treatments [2].

According to the most recent concept of carcinogenesis, direct experimental evidences support the hypothesis that tumors contain a small subpopulation(s) of cancer stem cells (CSCs) which have self-renewal and tumor initiation capacity. CSCs are also responsible for tumor heterogeneity, metastasis, resistance to anti-cancer therapies, and relapse [3,4]. CSCs are most commonly identified by cell surface markers. Colorectal cancer (CRC) stem cells express CD44, CD133, CD166, CD24, CD66c, CD29, the epithelial cell adhesion molecule, Musashi-1, aldehyde dehydrogenase-1 (ALDH1), and the leucine-rich-repeat containing G protein-coupled receptor 5 (LGR5) [5,6]. Previous studies have suggested that genetic alterations or microenvironment influences the appearance of CSC-like cells genotype [7,8]. The tumor suppressor protein p53 is frequently mutated in many types of human tumors, including CRC [9]. p53 protein plays a key role in stem cell biology, and regulates stem cell differentiation and self-renewal, quiescence, and multidrug resistance [10]. Recent studies on the direct and indirect links between CSCs and p53 have further solidified the importance of therapeutic targeting of p53 in CSCs. However, the mechanisms by which p53 regulates CSCs in CRC are poorly understood [11].

Melatonin, the major product of the pineal gland, is synthesized from tryptophan under the control of the enzymes arylalkylamine N-acetyltransferase (AA-NAT) and N-Acetylserotonin O-methyltransferase (ASMT) [12]. The presence of melatonin has also been detected in multiple extrapineal tissues in which melatonin-synthesizing enzymes have been identified [13]. Some of its functions are mediated through membrane (MT1 and MT2) or nuclear receptors (RZR/RORa) [14,15,16]. Melatonin also has other receptor-independent functions such as antioxidant and free radical scavenging [17,18]. Recently, the regulation of viability, proliferation, and differentiation of stem cells (SC) by melatonin has been proposed [19,20].

Melatonin plays an important role in cancer by inhibiting tumor growth and metastasis through different pathways, depending on the type of cancer studied and the dose of melatonin used [12]. In CRC, melatonin inhibits cell growth and induces apoptosis in vitro [21,22,23] and in vivo [24]. Recently, it has been reported that melatonin decreases CSCs and dysplastic injuries in colon tissue [24]. To date, there are no data linking loss of p53 function and melatonin synthesis or signaling in cancer, however some findings emphasize that activation of the p53 tumor-suppressor pathway, mediated by MT1 and MT2, may be a critical mediator in the anticancer activity of melatonin in CRC and breast cancer in vitro [25,26].

Previous data from our research team showed a decreased in MT1, MT2, and AA-NAT expression in CRC related to gender and invasion grade of tumors [27,28]. Herein, we investigate whether those expression decreases in melatonin synthesis and signaling genes are related to the expression of CSC markers in CRC and the implication of p53 status.

## 2. Results

### 2.1. Evaluation of Arylalkylamine N-acetyltransferase (AA-NAT), MT1, and MT2 Expression in Samples from Human colorectal cancer(CRC) with Different Status of p53

p53 sequencing analysis was successful in all 183 specimens. p53 mutations were detected in 76 patients (42%). Mutations are characterized in Table S1. The frequency of p53 mutations was different between tumor stages (stage I + stage II, and stage III + stage IV; *p* = 0.025) being higher in advanced stages of the disease (Table 1). That frequency was almost significant when considering the tumor differentiation grade (differentiated, moderately differentiated, and poorly differentiated; *p* = 0.095) but no differences were found between the anatomic location of the tumor (proximal, distal, rectal; *p* = 0.437).

**Figure 1:**
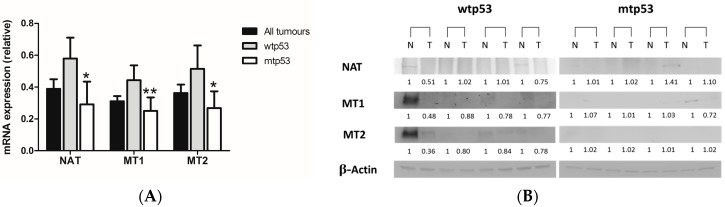
(**A**) mRNA expression of arylalkylamine N-acetyltransferase (AA-NAT), MT1, and MT2 in all patients, patients with wtp53 tumors and patients with mtp53 tumors. Data represent mean ± S.E.M. * *p* < 0.05 versus wtp53; ** *p* < 0.01 versus wtp53; (**B**) Protein expression of AA-NAT, MT1, and MT2 in normal (N) and malignant (T) colon samples in four patients with wtp53 and four patients with mtp53 tumors.

Next, we evaluated the expression of AA-NAT, MT1, and MT2 in our cohort of patients. In all patients, the expression in tumor samples versus normal mucosa was less than one for AA-NAT, MT1, and MT2 (0.39 ± 0.06, 0.31 ± 0.03, and 0.36 ± 0.05, respectively), which indicates that their expression decreased in tumor samples versus normal mucosa, as reported elsewhere [27,28,29]. Considering the p53 status, the expression of AA-NAT, MT1, and MT2 was lower in patients with mutant p53 (mtp53) than in patients with wild-type p53 (wtp53) (*p* < 0.05, *p* < 0.01, and *p* < 0.05, respectively) (Figure 1A). Protein expression of AA-NAT, MT1, and MT2 was higher in normal mucosa than in tumor samples in wtp53 tumors and weakly detected in mtp53 tumors (Figure 1B).

**Figure 2:**
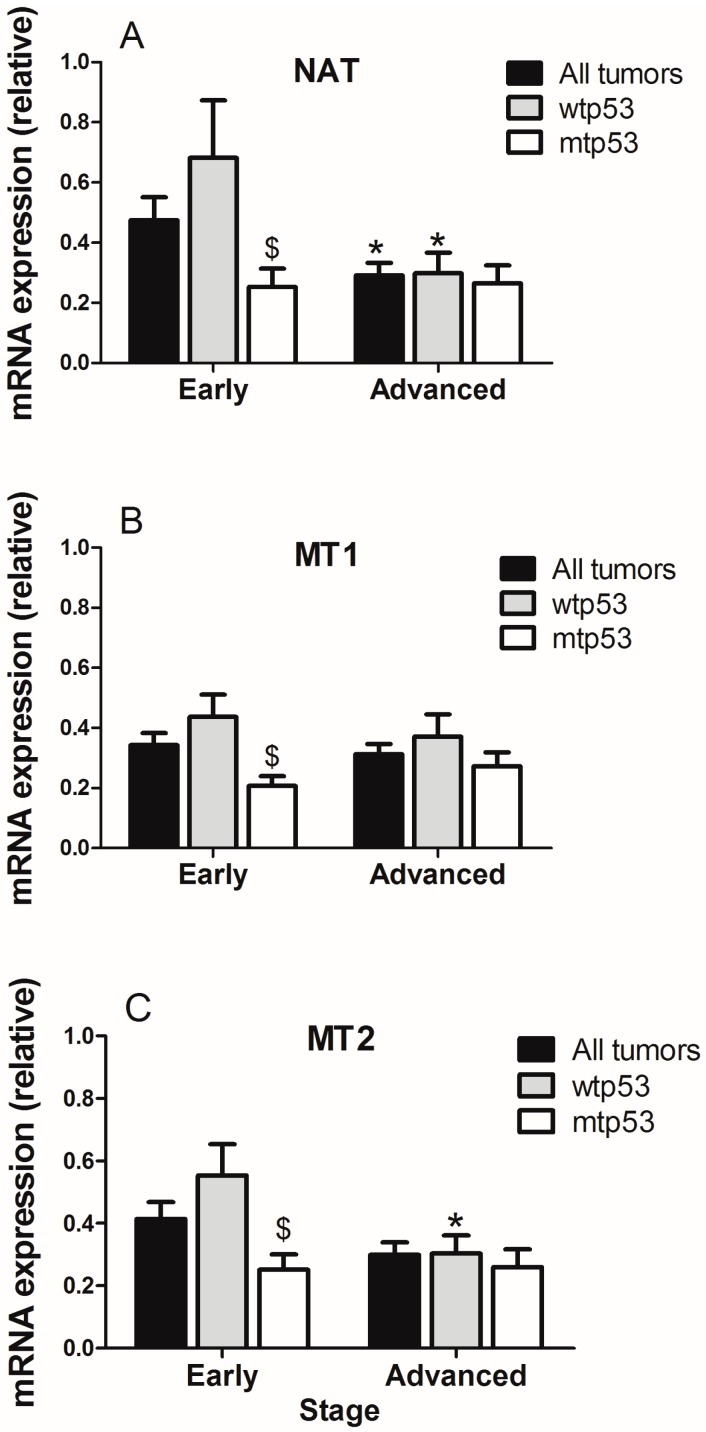
Expression of (**A**) AA-NAT; (**B**) MT1 and (**C**) MT2 in early stages (stage I + stage II) versus advanced (stage III + stage IV) colorectal tumors considering the status of p53. Data represent mean ± S.E.M. * *p* < 0.05 versus early; $ *p* < 0.05 versus wtp53.

When stratifying by tumor stage and p53 status, the expression of AA-NAT decreased significantly (*p* < 0.05) in advanced versus early stage tumors (Figure 2A), whereas the expression of MT1 and MT2 did not change in all patients (Figure 2B,C). In wtp53 tumors, differences in AA-NAT (*p* < 0.05) and MT2 expression levels (*p* < 0.05) were found between early and advanced tumors (Figure 2A,B). However, no significant changes in AA-NAT, MT1, and MT2 expression levels were found between early stage tumors and advanced ones in mtp53 samples (Figure 2). When comparing the gene expression at the same tumor stage, significant decreases in AA-NAT, MT1, and MT2 expression levels were found in early stage mtp53 tumors versus early stage wtp53 tumors (*p* < 0.05) (Figure 2A–C).

### 2.2. Evaluation of the Expression of Cancer Stem Cells (CSC) Markers in Samples from Human CRC with Different p53 Status

**Figure 3:**
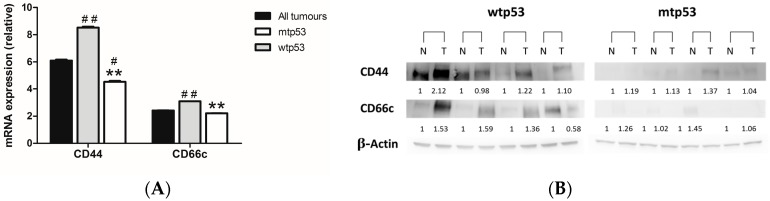
(**A**) mRNA relative expression of CD44 and CD66c in all patients, patients with wtp53 tumors and patients with mtp53 tumors. Data represent mean ± S.E.M. ** *p* < 0.01 versus wild-type; # *p* < 0.05 versus all patients; ## *p* < 0.01 versus all patients; (**B**) Protein expression of CD44 and CD66c in normal (N) and malignant (T) colon samples in four patients with wtp53 and four patients with mtp53 tumors.

We evaluated the expression of CD44 and CD66c as markers of CSC. In CRC, the expression of CD44 and CD66c are increased [6,30]. However, their regulation by p53 is not clear or has not described [6,31]. In our study, the relative expression of CD44 and CD66c was 6.10 ± 0.07 and 2.42 ± 0.01, respectively, which indicates higher expression in tumor samples than in normal mucosa. Interestingly, the expression of both markers was significantly higher (*p* < 0.01 in both cases) in wtp53 than in mtp53 tumors (Figure 3A). Protein expression of CD44 and CD66c was higher in tumor samples than in normal mucosa in wtp53 tumors and weakly expressed in mtp53 tumors (Figure 3B).

**Figure 4:**
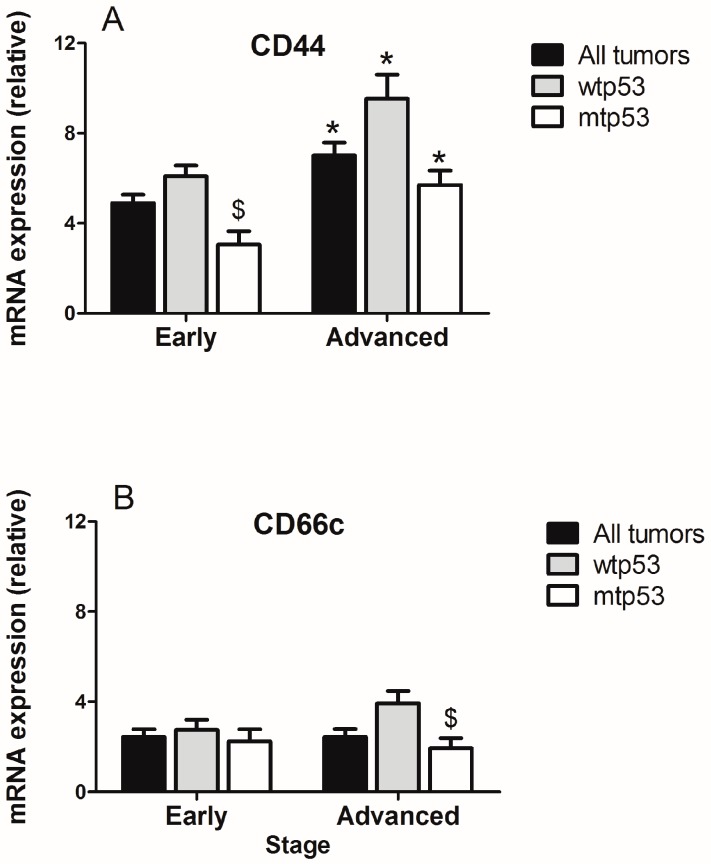
Relative expression of (**A**) CD44 and (**B**) CD66c in early stages (stage I + stage II) versus advanced (stage III + stage IV) colorectal tumors considering the status of p53. Data represent mean ± S.E.M. * *p* < 0.05 versus early; $ *p* < 0.05 versus wtp53.

The expression of CD44 increased in advanced tumors versus those at early stages and independently of the p53 status. However, CD66 expression was similar in both stages of the disease and was also independent of the p53 status (Figure 4). When comparing the expression of CD44 and CD66c at the same tumor stage, it was found that CD44 expression decreases in mtp53 versus wtp53 tumors in early stage tumors (*p* < 0.05) and that CD66c expression decreases in mtp53 versus wtp53 tumors in advanced stage of the disease (*p* < 0.05) (Figure 4A,B).

### 2.3. Correlation Between Markers of Cancer Stem Cells and Synthesis and Signaling Melatonin-Related Genes in Colorectal Cancer

**Figure 5:**
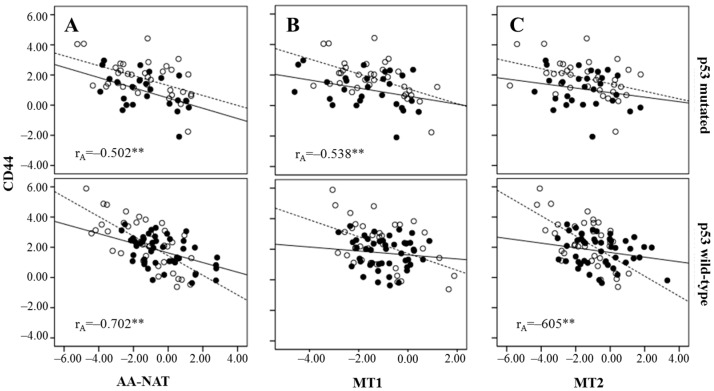
Correlation of CD44 expression and AA-NAT (**A**), MT1 (**B**), and MT2 (**C**) expression, stratifying by p53 status (wild-type or mutated) and in early stage tumors (—) and in advanced tumors (….). Pearson’s correlation coefficients: r_A_ (advanced tumors). ** *p* < 0.01.

The analysis of the expression of AA-NAT, MT1, MT2, CD44, and CD66c mRNA according to tumor stage and p53 status revealed a significant negative correlation between CD44 and AA-NAT in advanced stage tumors regardless of the p53 status, although a higher correlation was found in wtp53 tumors. CD44 expression also correlated with MT2 only in advanced stages and wtp53 tumors and with MT1 in advanced stages and mtp53 tumors (Figure 5).

**Figure 6:**
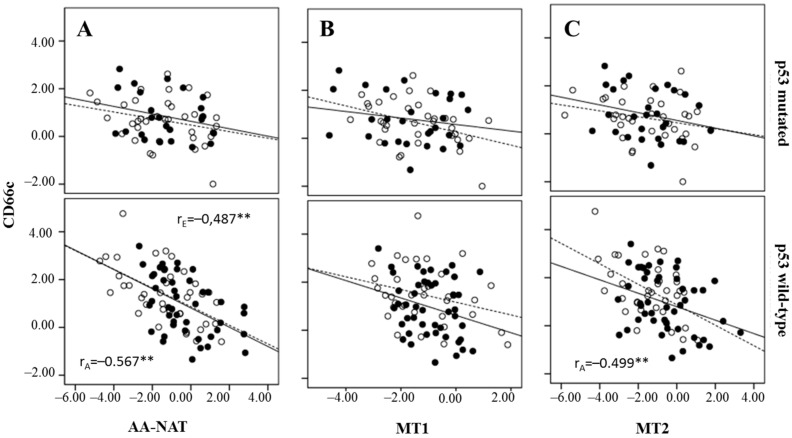
Correlation between CD66c expression and AA-NAT (**A**), MT1 (**B**) and MT2 (**C**) expression, stratification by p53 status (wild-type or mutated) and in early stage tumors (—) and in advanced tumors (….). Pearson’s correlation coefficients: r_E_ (early stage tumors) and r_A_ (advanced tumors). ** *p* < 0.01.

A negative correlation was also found between CD66c and AA-NAT in both early and advanced tumors, but only in wtp53 tumors, and between CD66c and MT2 in advanced and wtp53 tumors (Figure 6).

In our cohort of patients, 32.2% (*n* = 59) of tumors showed high expression of both CD44 and CD66c markers and were designated CD44_high_CD66c_high_, while 21.9% of tumors showed low expression of both markers and were designated CD44_low_CD66c_low_.

CD44_high_CD66c_high_ tumors correlated with low expression of both AA-NAT and MT2, in wtp53 tumors and advanced stages of the disease (Mantel-Haenszel test, *p* < 0.01 in both cases) (Table 2).

## 3. Discussion

At present, CSCs-based therapy represents a major challenge for the development of a novel generation of drugs and effective treatments in CRC, and other types of cancer. However, the mechanisms that regulate CSC renewal and carcinogenesis are not clear [32].

Very recent reports suggested that the oncostatic effects of melatonin could be related to its cytotoxicity induced in CSCs of the brain [33,34], glioblastoma [35], and breast tumors [36]. In CRC, melatonin reduced the CSC pool and the dysplastic injuries induced by constant light and carcinogen treatments in a model of rats [24]. According to this, we found that AA-NAT, MT1, and MT2 negatively correlate to CD44 and CD66c in CRC. In fact, melatonin regulates key pathways involved in intestinal mesenchymal and CSC differentiation, such us the Wnt/β-catenine, BMPs, and Notch pathways [19,37,38,39,40].

Several studies have delineated the effects of p53 loss and p53 mutation on the CSC pool [11]. Loss of wtp53 function enriches putative colon CSCs, and restoration of wild type p53 in p53-null HCT116 cells eliminated the putative CSC populations [41]. The combination of 5-fluorouracil plus p53 pathway restoration is associated with depletion of p53-deficient or mutant p53-expressing putative colon CSCs [42]. Other studies showed that HMGA1 silencing restores normal stem cells (SC) characteristics in colon CSCs by increasing p53 levels [43]. It has been recently demonstrated that p53 transcriptionally represses CD44 protein expression in both normal and tumor-derived mammary epithelial and lung tumor cells [44]. However, loss p53 function is not associated with elevated CD44 expression in CCR [31]. According to this, our results showed higher expression of CD44 in wtp53 than in mtp53 tumors. CD66c expression also decreases in mtp53 tumors versus those bearing wtp53.

Melatonin has been shown to regulate the expression of both p53 and p21 [45] and to increase p38-mediated p53 phosphorylation in cancer cells and the p53-dependent DNA damage response [26,46] trough MT1 and MT2 [25]. A decreased expression of AA-NAT, MT1, and MT2 in CCR mtp53 tumors was found in our study. Our results are consistent with previously published data from our research group [27,28], with a lower expression of AA-NAT and MT2 found in advanced rather than in early stages of the disease. However, this was found in wtp53 tumors but not in mtp53 tumors. These results indicate that a loss of p53 could lead to decreased expression of AA-NAT and MT2 in CRC, and that p53 could regulate their expression, or vice versa. In fact, melatonin is a molecule with important antioxidant properties [17,18] and a decrease in its synthesis and signaling could lead to increased oxidative stress in the colonic tissue [47], which in turn could produce p53 mutations [48]. Our results are also consistent with those reported by Nemeth et al., who found decreased expression of the melatonin receptor 1 in human colorectal adenocarcinomas [29]. Recently, Ziolko et al. also studied the profile of the melatonin MT1 receptor gene in CRC, and they reported a significant increase in its levels of expression in cancerous versus non-cancerous tissue [49]. These discrepancies could be due to the low number of patients included in these two studies (24 and 39, respectively), while our cohort includes 184 patients.

There is no consensus about the origin of CSCs in CRC. Although, under the CSC hypothesis the origin of the tumor is not necessarily a SC, this could be the case in the intestine, a tissue where the differentiated cells are renewed every 2–7 days and whose functional unit, the Lieberkühn crypts, is underlyed by mesenchymal stem cells (MSCs). Regardless of their origin, CSCs are of considerable clinical significance, having been shown to be more resistant to radiotherapy and chemotherapy than other tumor cells. This is a feature shared with normal SCs, which present various strategies to resist chemotherapy, including cellular quiescence and the expression of proteins capable of eliminating drugs from the cytoplasm, such as ATP-binding cassette (ABC) transporters and multidrug resistance (MDR) proteins. [50]. During the process of transformation to CSCs, decreased expressions of genes related to melatonin synthesis and signaling pathways could take place. The fact that we have found a negative correlation between AA-NAT, MT1, and MT2 with CD44 and CD66c in advanced stages of the disease, in our cohort of patients, reinforces this hypothesis, although, more studies are necessary to confirm it. Further, we found that those correlations are higher in wtp53 tumors, which indicates that a functional p53 protein is necessary for the interrelation between melatonin-related genes and CSC markers in CRC to exist.

Personalized medicine involves the use of genetic information about an individual patient to select or optimize the patient’s preventive and therapeutic care. Despite the success of new treatments, most patients eventually progress due to the presence of intra- and inter-tumor heterogeneity conferring resistance. Factors contributing to heterogeneity include the presence of CSC subpopulations, among others [51]. P53 is a very important factor involved in the development of CSC subpopulations in CRC [11,41,42,43]. The presence of p53 mutations induces resistance in cultured CRC cells and is indicative of poor survival in patients after 5-fluorouracil treatment [52,53], the gold standard in CRC chemotherapy treatment that, depending on the tumor stage, is combined with other drugs [54]. Melatonin is able to increase the cytotoxicity of 5-fluorouracil in cultured cells and in patients with advanced (metastatic) CRC [55,56]. Here we show a possible mechanism of this melatonin effect and the possibility of using it in combination with 5-fluorouracil in patients with mtp53 resistant to treatment with 5-fu alone. The five-transmembrane glycoprotein CD133 is one of the first colon CSC markers identified [57] and its use as a CSC marker has been controversial since then [58]. The selection of colon cancer cells based on the positivity for AC133, an epitope on the CD133 protein, allows for the identification of tumorigenic and clonogenic cell populations [59]. On the other hand, CD133^+^ as well as CD133^−^ metastatic colon cancer cells were shown to be able to form new tumors and their expression does not seem to be restricted to the stem cell compartment, which indicates that use of CD133 as a CSC marker is questionable [60,61,62]. Since colon cancer cells sorted for CD44_high_ or CD66c_high_ displayed high tumorigenicity [5,6], we used these CSCs markers in our study. CD44_high_CD66c_high_ tumors also correlated with low expression of AA-NAT and MT2 in wtp53 tumors in advanced stages of the disease, which is indicative of the important role of melatonin and it’s signaling through MT2 in CRC.

In conclusion, in view of the results from this study, melatonin could be considered a therapeutic approach in patients with advanced CRC and with tumors bearing wtp53, decreasing, at least in part, the resistance to commonly used therapies [34]. In the near future, the oncology community will need to validate this possibility and make certain that patients who are candidates for melatonin therapy undergo the appropriate testing and treatment.

## 4. Materials and Methods

### 4.1. CRC Human Samples

The Andalusian Tumor Bank Network (RBTA) provided the samples used. The Ethics Committee of San Cecilio University Hospital approved (Ethical Committee of Clinical Research of Granada; proyect code: PI-067/2013; date of approval: 01/24/2014) the study and all patients gave written informed consent for the use of samples in biomedical research. All specimens (viable tumor tissues and adjacent normal tissues) were dissected immediately and fresh-frozen in Tissue-Tek1 (Optimal Cutting Temperature Compound, Sakura Finetek Europe B.V., Zoeterwoude, The Netherlands) by standard methods. AA-NAT, MT1, MT2, CD44, CD66c, and Ubiquitin C (UBC) mRNA were evaluated in the tumor tissue and adjacent normal mucosa of 183 patients (mean age 71 ± 9 years) who underwent surgery for primary sporadic CCR (Table S2). In relation to the tumor staging (classified according to TNM nomenclature), the samples were grouped in early (stage I + stage II) and advanced (stage III + stage IV) stages because no significant results were obtained in each group separately. DNA was also extracted to evaluate the status of p53.

### 4.2. RNA Isolation and cDNA Synthesis

Total RNA from tissue samples was prepared using the TRIzol reagent (Invitrogen, Carlsbad, CA, USA). The amount of total RNA was determined by UV spectrophotometry, and RNA integrity was assessed by agarose gel electrophoresis. First-strand cDNA was prepared by reverse transcription with oligo-dT primers from 2 μg of total RNA in 50 μL of total volume using a commercial cDNA synthesis kit (AccuScriptTM High Fidelity 1st Strand cDNA Synthesis Kit, Stratagene, San Diego, CA, USA).

### 4.3. Real-Time PCR (RT-PCR)

About 5 μL of the cDNA was amplified for 40 cycles with specific primers for AA-NAT, MT1, MT2, CD44, CD66c, and UBC (Table S3). PCR reactions containing SYBR-green were performed using the Mx3000P QPCR System (Stratagene, Austin, TX, USA). UBC was used as housekeeping to normalize mRNA levels. Standard curves were generated for each target gene by plotting *C*_t_ values versus log cDNA dilution. PCR products were verified by a melting profile and, agarose gel electrophoresis to rule out nonspecific PCR products and primer dimers.

### 4.4. DNA Extraction

Genomic DNA was isolated from tissue samples using the QIAamp ADN mini Kit (Qiagen, Hilden, Germany) according to the manufacturer’s instructions. The DNA was quantified on a NanoDrop ND-1000 spectrophotometer (Implen GmbH, Munich, Germany) and analyzed by agarose electrophoresis. The 260/280 ratios ranged from 1.7 to 2.0, yet gel electrophoresis indicated a generally acceptable quality for these samples.

### 4.5. Sequencing of p53 Mutations

The samples were analyzed for TP53 mutations in exons 2–10 by PCR using specific primers (Table S4). Automated DNA sequencing of PCR products was performed using a 3130XL Genetic Analyzer and then assessed with Sequence Scanner v1.0 software (Applied Biosystems, Foster City, CA, USA).

The detected mutations were confirmed by two independent amplifications and reactions. The IARC p53 database (http://www.p53.iarc.fr/), which describes the activity of each p53 protein encoded by a single-nucleotide point mutation, was consulted [63]. TP53mutants are considered to have a median transcriptional activity >75% were classified as wild type for the calculation. A median transcriptional activity <75% was considered partially functional and was classified as mutant for the calculation [64].

### 4.6. Western Blotting

Tissues from patients were homogenized in ice-cold lysis buffer (50 mM Tris-HCl pH 7.4, 150 mM NaCl, 0.1% SDS, 1% Igepal C, 0.5% sodium deoxycholate, 1 mM Na_3_VO_4_, 10 mg/mL leupeptin, 2 mg/mL aprotinin, and 1 mg/mL pepstatin, 1 mM PMSF, 10 nM NaF) on ice for 45 min and clarified by centrifugation at 16,000× *g* for 2 × 30 min. Fifty micrograms of protein were loaded into 12% SDS-polyacrylamide gels. Subsequently, the proteins were transferred to PVDF filters and the blots were probed with appropriate antibodies for MT1 (Santa Cruz Biotechnology, Inc., Dallas, TX, USA), MT2 (Santa Cruz Biotechnology, Inc.), AA-NAT (Santa Cruz Biotechnology, Inc.), CD44 (Santa Cruz Biotechnology, Inc), CD66c (Santa Cruz Biotechnology, Inc.), and β-actin (Santa Cruz Biotechnology, Inc.). Immunoreactivity was assessed on an enhanced electrochemoluminescence autoradiography system (Amersham Biosciences, Buckinghamshire, UK).

### 4.7. Statistical Analysis

mRNA levels of genes in tumor tissues were normalized using their levels in normal mucosa from the same patient and were compared by the paired Student’s *t* test. Pearson’s correlation was used to express the results of the correlation analyses. Bivariate analysis was performed using the χ^2^ test and the Fisher’s exact and Mantel–Haenszel tests. Cut-off value between low and high expression level was set at the median mRNA expression level for each of the genes analyzed. *p* values lower than 0.05 were considered significant. All statistical calculations were performed using SPSS software version 15.0 for Windows (SPSS Inc., Chicago, IL, USA).

## Figures and Tables

**Table 1:** Prognostic covariates.

Covariates	TP53 ^a^ Wild-Type % (*n*)	TP53 Mutant % (*n*)	*p* ^b^
Gender			
Male	62.0 (66)	63.2 (48)	0.893
Female	38.0 (41)	36.8 (28)	
Differentiation Grade			
Well differentiated	16.8 (18)	35.5 (27)	0.095
Moderately differentiated	69.1 (74)	51.3 (39)	
Poorly differentiated	14 (15)	13.2 (10)	
Stage			
Stage I + II (Early)	59.8 (64)	33.3 (32)	**0.025**
Stage III + IV (Advanced)	40.2 (43)	57.9 (44)	
Location			
Proximal	47.7 (51)	38.2 (29)	0.437
Distal	47.7 (51)	56.6 (43)	
Rectal	4.6 (5)	5.2 (4)	

^a^ P53 gene; ^b^ Significant associations are shown in bold (*p* < 0.05).

**Table 2:** Relationship between cancer stem cell CSC markers and melatonin synthesis and signaling genes according to p53 status and progression of the disease.

	**p53 Wild-Type**					
**Gene**	**Early Stages**			**Advanced Stages**		
	**CD44_low_CD66c_low_*n* (%)**	**CD44_high_CD66c_high_*n* (%)**	***p*^a^**	**CD44_low_CD66c_low_*n* (%)**	**CD44_high_CD66c_high_ n (%)**	***p*^a^**
**NAT ^b^**			ns			0.045
Low	7 (63.6)	17 (89.5)		4 (40.0)	16 (80.0)	
High	4 (36.4)	2 (10.5)		6 (60.0)	4(20.0)	
**MT1**			ns			ns
Low	11 (91.7)	19 (95.0)		7 (77.8)	18 (90.0)	
High	1 (8.3)	1 (5.0)		2 (22.2)	2 (10)	
**MT2 ^b^**			ns			
Low	8 (66.7)	17 (85.0)		4 (44.4)	20 (100.0)	
High	4 (33.3)	3 (15.0)		5 (55.6)	0 (0.0)	
	**p53 Mutated**					
**Gene**	**Early Stages**			**Advanced Stages**		
	**CD44_low_CD66c_low_*n* (%)**	**CD44_high_CD66c_high_*n* (%)**	***p*** **^a^**	**CD44_low_CD66c_low_ n (%)**	**CD44_high_CD66c_high_*n* (%)**	***p*** **^a^**
**NAT ^b^**			ns			ns
Low	6 (75.0)	4 (80.0)		4 (40.0)	10 (83.3)	
High	2 (25.0)	1 (20.0)		6 (60.0)	2 (16.7)	
**MT1**			ns			ns
Low	9 (100.0)	5 (71.4)		7 (77.8)	11 (100.0)	
High	0 (0.0)	2 (28.6)		2 (22.2)	0 (0.0)	
**MT2 ^b^**			ns			ns
Low	7 (77.8)	6 (85.7)		6 (66.7)	9 (81.8)	
High	2 (22.2)	1 (14.3)		3 (33.3)	2 (18.2)	

^a^ Bivariate analysis: *p* calculated by *χ*^2^ test or Fisher’s test; ^b^ Mantel–Haenszel test: *p* ≤ 0.01.

## Supplementary Materials

Supplementary materials can be found at www.mdpi.com/1422-0067/18/6/1251/s1.

## Abbreviations

ALDH1	Aldehyde dehydrogenase-1
AA-NAT	Arylalkylamine *N*-acetyltransferase
ASMT	*N*-Acetylserotonin *O*-methyltransferase
CRC	Colorectal cancer
CSCs	Cancer stem cells
HGVS	Human Gene Variation Society
Lgr5	Leucine-rich-repeat containing G protein-coupled receptor 5
MSCs	Mesenchymal stem cells
Mtp53	Mutated p53
Wtp53	Wild-type p53
