# Supplementary material for: AA-NAT, MT1 and MT2 Correlates with Cancer Stem-Like Cell Markers in Colorectal Cancer: Study of the Influence of Stage and p53 Status of Tumors
Source: Int J Mol Sci. 2017 Jun 11;18(6):1251. doi: 10.3390/ijms18061251 (PMC5486074; doi:10.3390/ijms18061251)
Supplement: Supplementary file 1 [file ijms-18-01251-s001.pdf]

Supplementary Table 1. P53 mutations.

Type of TP53 mutation	Number of patients with the mutation	Exon
c.375G>A (p?)	1	4
c.404G>T (p.C135F)	2	5
c.422G>A (p.C141Y)	1	5
c.423C>G (p.C141W)	1	5
c.438G>A (p.W146X)	1	5
c.455C>T (p.P152L)	2	5
c.481G>T (p.A161S)	1	5
c.488A>G (p.Y163C)	4	5
c.510G> (p.T170X)	1	5
c.517G>T (p.V173L)	2	5
c.515T>G (p.V172G)	1	5
c.520A>T (p.R174W)	1	5
c.524G>A (p.R175H)	8	5
c.526_52del (p.C176X)	1	5
c.527G>T (p.C176F)	1	5
c.536A>G (p.H179R)	1	5
c.584T>C (p.I195T)	1	6
c.632C>T (p.T211I)	1	6
c.637C>T (p.R213X)	5	6
c.659A>G (p.Y220C)	1	6
c.672+2T (p ?)	1	Intron 6[b]
c.713G>A (p.C238Y)	2	7
c.733G>A (p.G245S)	4	7
c.734G>A (p.G245D)	2	7
c.742C>T (p.R248W)	2	7
c.743G>A (p.R248Q)	4	7
c.745A>T p.R249W	1	7
c.757_75del (p.T253X)	1	7
c.775G>T (p.D259Y)	1	7
c.783-1G (p?)	1	Intron 7[b]
c.797G>A (p.G266E)	1	8
c.811G>A (p.E271K)	2	8
c.814G>A (p.V272M)	2	8
c.817C>T (p.R273C)	4	8
c.818G>A (p.R273H)	5	8
c.818G>C (p.R273P)	1	8
c.844C>T (p.R282W)	2	8
c.853G>A (p.E285K)	1	8
c.919+1G (p?)	1	Intron 8[b]
c.904G>T (p.G302W)[a]	1	8

To report mutations, we followed the recommendations provided by the Human Gene Variation Society (HGVS).

[a]Mutation was considered and calculated as wild type due to transactivation activity > 75%.

[b]Three mutations, though located in the introns, had a predicted effect on splicing and were classified as mutant

Supplementary Table 2. Characteristics of the patients included in the study

CHARACTERISTIC	N (%)
**Gender**	
Female	110 (60)
Male	73 (40)
**Location**	
Colon	174 (95)
Rectum	9 (5)
**Histological type**	
Well differentiated	45 (24.4)
Moderately differentiated	113 (61.6)
Poorly differentiated	25 (14.0)
**pTMN Stage**	
Stage I	23 (12.4)
Stage II	73 (39.9)
Stage III	74 (40.4)
Stage IV	13 (7.3)

Supplementary Table 3. Primers used to determine AA-NAT, MT1, MT2, CD44 and CD66c expression.

Gene	Forward	Reverse	Product size (bp)
AA-NAT	tccctgccagtgagtttcgctgcctcac	tgtcccagagcgagccgatgatgaaggc	206
MT1	ttgtcctttttgccatttgctgggctcctc	gtcatcagtggagacggtttccatttaacc	289
MT2	gtggtgtttgtgatctttgccatctgctgg	agcatctgcctggtgctgcacaccaatgat	321
CD44	gctttcaatagcaccttgcccacaatgg	aaagaggtcctgtcctgtccaaatcttcc	594
CD66c	acccagtcaccctgaatgtcctctatgg	gacaggagcacttccagagactgtgatc	293
UBC	tgggatgcaaatcttcgtgaagaccctgac	accaagtgcagagtggactctttctggatg	213

Supplementary Table 4. Primers used to analyze p53 mutations

Exons	Primers		Product Size (pb)
	Sense	Antisense	
**2-4**	agctgtctcagacactggcatggtgttgg	cactgacaggaagccaaagggtgaagagg	840
**5-6**	gttgctttatctgttcacttgtgccctgac	tagggaggtcaatatagcagcaggagaaag	548
**7-9**	cagcctgggcgacagagcgagattccatc	aaccaggagccattgtctttgaggcatcac	987
**10**	tacttgaagtgcagtttctactaaatgcatg	Aggaagactaaaaaaatgtctgtgcagggc	393
